# A case of chronic hepatitis B merged with acute fatty liver of pregnancy with severe coagulopathy

**DOI:** 10.1186/s40981-018-0219-5

**Published:** 2019-01-07

**Authors:** Risa Fukushima, Kotoe Kamata, Fumiko Ariyoshi, Masashi Yanaki, Minoru Nomura, Makoto Ozaki

**Affiliations:** Department of Anesthesiology, Tokyo Women’s Medical University, 8-1 Kawada-cho, Shinjuku-ku, Tokyo, 162-8666 Japan

**Keywords:** Acute fatty liver of pregnancy, Hepatitis B, Parturient, Hypothermia

## Abstract

**Background:**

Acute fatty liver of pregnancy (AFLP) is a life-threatening disorder, and its relevance to viral hepatitis B (HB) remains unknown. This case presents an initial experience of treating a patient with HB progressing to AFLP throughout pregnancy; anesthesiologists should also recognize its clinical feature for perioperative management.

**Case presentation:**

A 28-year-old parturient was diagnosed as chronic HB (CHB) at 21 weeks gestation. Liver and kidney dysfunction appeared rapidly at 34 weeks gestation, suspected as acute exacerbation of either CHB or AFLP. Emergency cesarean section was carried out, after which maternal disseminated intravascular coagulation and hypothermia persisted. With multidisciplinary management, the patient and infant were discharged on postpartum days 64 and 12, respectively.

**Conclusions:**

Active CHB develops into AFLP. Antiviral therapy should be considered for parturient patients with CHB, particularly for those with high viral load. The most favorable outcome is prompt and accurate diagnosis to establish suitable termination method.

## Background

Acute fatty liver of pregnancy (AFLP) is rare liver disorder that occurs in approximately 5 cases per 100,000 maternities [1]. Since the development of forced delivery techniques and intensive transfusion therapy, maternal mortality rate has dramatically decreased from 75 to 1.8% whereas neonatal mortality remains as high as 104 deaths per 1000 deliveries [1, 2]. Factors frequently reported as being associated with AFLP include elderly primigravida, multiple gestations, preeclampsia, and male fetus [1, 3]. AFLP etiology has been suggested as defective fatty acid oxidation in the fetus, but a method for prevention has not yet been established [3–5]. On the other hand, fulminant hepatitis (FH), a type of viral infection, shares a number of similar clinical features. Both diseases occur mainly in late pregnancy and manifest with jaundice, liver dysfunction, and rapidly progressing severe coagulopathy [3, 6]. In this case, chronic hepatitis B (CHB) was merged with AFLP, requiring emergency cesarean section. As there have been no reports examining such cases in term of the attention and preparatory points for anesthesia, our report also considers these aspects.

## Case presentation

A Chinese 28-year-old female, previously a healthy 49 kg, Gravida 2 Para 0 at 21 weeks gestation, was diagnosed with liver dysfunction during a routine health checkup (Table 1). She was referred to our institution due to suspected hepatitis B (HB). Detailed examination revealed IgM anti-HBc was negative. However, excess levels of HBe-antigen (1017.6 S/CO), HBs-antigen (> 250 S/CO), and HB virus DNA (> 9.0 Logcopies/mL) indicated she suffered from CHB. HB virus genotype was proved B. Intravenous monoammonium glycyrrhizinate therapy, started at 25 weeks gestation, was effective and decreased alanine aminotransferase (ALT) levels to within normal range. However, at 35 weeks and 4 days gestation, the patient required hospitalization when she presented severe malaise with elevated transaminase levels and coagulopathy (Table 1). She was diagnosed with acute exacerbation of CHB and was started on 300 mg of oral tenofovir (300 mg, daily) and intravenous vitamin K (20 mg, daily). The next morning, she presented systemic edema. The following afternoon she complained of abdominal pain and nausea. Transabdominal ultrasonography showed ascites and a fatty liver, suggesting AFLP. At this time, she showed blood pressure of 117/68 mmHg, pulse of 86 beats/min, and body temperature of 36.7 °C. Considering the progressive somnolence and sufficient gestational age without cervical dilatation, emergency cesarean section was selected, before referral to anesthesiologists for a detailed preoperative evaluation. The Swansea Criteria, which defines AFLP at 6 points or more [1, 4, 7], scored at least 7 points at this time (abdominal pain, elevated bilirubin, ascites or bright liver on ultrasound scan, elevated transaminase, elevated ammonia, coagulopathy, and vomiting were positive; hypoglycemia, elevated uric acid, and liver biopsy were not examined; polydipsia or polyuria, encephalopathy, leukocytosis, and renal impairment were negative). To control coagulopathy, 24 units of fresh frozen plasma (FFP) and 3000 units of antithrombin formula were administrated according to the obstetricians’ estimations. Additionally, 20 units of platelet concentrate (PC) were given due to severe thrombocytopenia (Table 1; 35 5/7 weeks, 5 p.m.). The patient’s vital signs following preoperative blood transfusions were blood pressure of 97/46 mmHg, pulse rate of 86 beats/min, body temperature of 36.0 °C, and peripheral oxygen saturation (SpO_2_) of 97% in room air. The patient was classified as American Society of Anesthesiologists Physical Status Class 3E. The fetus showed reassuring status. In consideration of the severe coagulopathy as well as possible leukoencephalopathy, we avoided using neuraxial block despite the massive preoperative transfusion.

**Table 1:** Blood sampling data

	Reference range	21 weeks	28 weeks	35-4/7 weeks	35-5/7weeks 9 a.m. /5 p.m.	Pre-operative (35-6/7) 1 a.m.	Post-operative	POD 4	POD 7	POD 62	Post-operative 1 year
T-Bil (mg/dL)	0.2–1.2	1.2	1.3	4.6	5.7/NE	NE	3.6	13.5	19.0	4.6	0.9
ALT (U/L)	6–30	214	29	41	44/NE	NE	23	28	28	16	20
ALB (g/dL)	3.8–5.1	3.5	NE	NE	2.2/NE	NE	2.2	3.2	3.5	3.5	4.1
NH_3_ (μ/dL)	12–66	NE	NE	NE	132/NE	NE	NE	70	65	56	NE
Cre (mg/dL)	0.48–0.79	0.38	0.44	0.63	0.68/NE	NE	0.62	0.60	2.29	0.51	0.53
UA (mg/dL)	2.4–5.9	2.6	NE	NE	NE/NE	NE	3.8	1.8	NE	NE	3.6
PT (%)	70–130	90.9	NE	31.6	29.1/27.0	56.7	47.3	29.3	37.0	56.0	NE
APTT (sec)	25.5–39.5	34.6	NE	NE	NE/64.2	42.0	61.2	> 150	42.9	NE	NE
AT-III (%)	80–130	NE	NE	NE	NE/< 10	99	60	52	88	NE	NE
Fib (mg/dL)	150–350	NE	NE	NE	NE/< 50	142	146	199	185	NE	NE
WBC (/μL)	4000–8600	8050	7950	9330	10,180/7310	6000	5950	11,290	18,480	1880	2320
Hb (g/dL)	12.0–16.0	11.7	10.1	11.1	11.7/9.4	8.8	7.4	9.1	9.3	10.5	8.4
PLT (K/μL)	150–350	167	165	62	59/50	37	52	75	35	41	66

*NE* not examined, *T-Bil* total bilirubin, *ALT* alanine aminotransferase, *ALB* albumin, *NH*_*3*_ ammonia, *Cre* creatinine, *UA* uric acid, *PT* prothrombin time, *APTT* activated partial thromboplastin time, *AT-III* antithrombin, *Fib* fibrinogen, *WBC* white blood cell, *Hb* hemoglobin, *PLT* platelet count

Rapid sequence induction with thiopental 250 mg, fentanyl 100 μg, and suxamethonium 50 mg was selected. The trachea was easily intubated. General anesthesia was maintained with 65% nitrous oxide and 1.0% sevoflurane until delivery. Though fluid-warming devices and a forced-air warmer were prepared, patient rectal temperature dropped to 34.8 °C after induction. A female infant of 2773 g with good APGAR scores (8 and 9 at 1 and 5 min, respectively) was delivered 7 min after anesthesia induction. The mother showed hypoglycemia of 65 mg/dL, which was corrected by 10 g of dextrose. Despite appropriate uterine contraction, additional transfusion was required to maintain hemostasis during the operation. Four units of packed red blood cell (RBC), 5 bags of cryoprecipitate, 10 units of PC, and 800 mL normal saline were administered on the intraoperative anesthesiologist’s decision. Estimated blood loss, ascites, amniotic fluid, and urine output were 2020 mL, 300 mL, 450 mL, and 650 mL, respectively. The total duration of surgery was 69 min, and anesthesia time was 81 min. Prolonged hypothermia was recorded as her rectal temperature had dropped to 34.3 °C by the end of the surgery.

After cesarean section, the patient was transferred to the intensive care unit (ICU) with tracheal intubation. Vaginal hemorrhage persisted for 12 h, despite transfusion of 18 units of packed RBCs 16 units of FFP and 10 units of PC. Eventually, active hemorrhage was controlled by uterine artery embolization. She was extubated on postoperative day (POD) 2 and was well orientated with stable vital status. At one point, liver transplantation was planned, due to that the Model for End-stage Liver Damage score for acute disseminated intravascular coagulation (DIC) and liver dysfunction [8] on POD 4 was 24 points; however, 4 days of therapeutic plasma exchange (POD 3–6) and 6 days of hemodiafiltration (POD 3–8) were highly effective. Glycerin fructose was used for 9 days (on POD 2–10) because of cerebral edema, but she showed no neurological sequelae. Neither infection, sepsis, major respiratory complications, nor pancreatitis was remarked. Liver biopsy was not performed in consideration of coagulopathy. The patient was discharged from ICU and hospital on POD 13 and 64, respectively. HB virus vaccination and HB immunoglobulin given at birth prevented vertical transmission of HB, and the infant did not show any developmental delay. The infant was discharged from the hospital on postpartum day 12. For 1-year follow-up after discharge, ALT level of the patient has been kept in normal range and her HBV-DNA level as less than 3.0 Logcopies/mL (Table 1).

## Discussion

Viral hepatitis patients generally have higher levels of serum transaminases, with values exceeding 1000 U/L. The level of uric acid is rarely elevated in FH patients, and signs of preeclampsia are absent in viral hepatitis [3]. Compared to AFLP, multiorgan failure is more likely to coexist with viral hepatitis. On the other hand, incidences of hypercreatinemia, DIC, and digestive tract hemorrhage are less common in patients with viral hepatitis than in those with AFLP [9]. It is also suggested that AFLP patients tend to show hypoglycemia [4]. These clinical features may be supportive in distinguishing between viral hepatitis, FH and AFLP. Termination is selected as a basic treatment for both AFLP and FH; however, especially in early preterm FH cases, careful observation with antiviral drug therapy may be an alternative in consideration of neonatal outcomes. In fact, as both AFLP and FH commonly reveal in the late pregnancy [1, 3, 6], accurate differential diagnosis is not always as important as it was for this case. Our judgment to carry out an urgent cesarean section, without accurate diagnosis, can be considered reasonable and necessary, given the sufficient gestational age, severity of liver dysfunction and reassuring fetal status. Moreover, antiviral agent therapy has been commonly utilized in pregnant cases. Tenofovir, a kind of nucleoside reverse transcriptase inhibitor that works by decreasing the amount of HB virus in the blood, has been confirmed as safe for use with fetuses, as evidenced by its level B status given by the Food and Drug Administration [10]. The latest report recommended the use of antiviral drugs to a pregnant patient with a high viral load (100,000–200,000 IU/mL) during their third trimester [10]. This treatment not only benefits the mother, but also the fetus by preventing mother-to-child transmission. In addition, HB virus vaccination and HB immunoglobulin are recommended for neonates as in this case [10]. More in-depth research is expected to determine if the levels of transaminase and HBV-DNA and virus genotype are related to AFLP and if antiviral agents are able to prevent AFLP onset.

Regarding anesthesia, some reports have shown the key to perioperative management of AFLP to be as follows: early diagnosis, prompt termination, strict fluid management, correct coagulopathy, and care for hypoglycemia [1, 3]. These warnings were of course applied for the current case, but body temperature proved difficult to control. There are few reports concerning perioperative body temperature in AFLP patients. Although mild hypothermia control of 32 to 35 °C is sometimes carried out in hepatic encephalopathy patients, hypothermia may cause coagulopathy [11, 12]. As the relationship between infection or hemorrhage and body temperature are still under investigation, we concluded normothermia to be acceptable, providing hepatic encephalopathy was not present. During surgery, we aimed to keep the patient’s body temperature over 36 °C; however, we failed to achieve this, despite using fluid-warming devices and a forced-air warmer. Preoperative temperature control would have been better as the massive transfusion prior to operation could be the cause of hypothermia. Close-knit integration and sufficient communication with obstetric and clinical staff on the ward are also required. It is sometimes difficult to keep core temperature in severe liver dysfunction status patients stable due to dilation of peripheral blood vessels.

## Conclusions

Active CHB develops into AFLP. Antiviral therapy should be considered for pregnant patients with CHB, especially in the cases with high viral load. Prompt and accurate diagnosis is favorable in order to evaluate how urgent termination should be carried out. However, in cases of late preterm period without cervical dilatation, elected urgent cesarean section should be selected. If normothermia is to be maintained during surgery, perioperative management on the ward before admission to the operating room is necessary.

## Abbreviations

AFLP: Acute fatty liver of pregnancy
ALT: Alanine aminotransferase
CHB: Chronic hepatitis B
DIC: Disseminated intravascular coagulation
FFP: Fresh frozen plasma
FH: Fulminant hepatitis
HB: Hepatitis B
ICU: Intensive care unit
PC: Platelet concentrate
POD: Postoperative day
RBC: Red blood cell
SpO_2_: Pulse oximetry

## Authors’ information

RF is an assistant professor in the Department of Anesthesiology at Tokyo Women’s Medical University. KK is an assistant professor in the Department of Anesthesiology at Tokyo Women’s Medical University. FA is an assistant professor in the Department of Anesthesiology at Tokyo Women’s Medical University. MY is an assistant professor in the Department of Anesthesiology at Tokyo Women’s Medical University. MN is a professor in the Department of Anesthesiology at Tokyo Women’s Medical University. MO is a professor in the Department of Anesthesiology at Tokyo Women’s Medical University.
